# Circulating multiple metals and mortality after myocardial infarction: incremental value beyond GRACE

**DOI:** 10.1016/j.ajpc.2026.101548

**Published:** 2026-03-15

**Authors:** Minmin Wu, Junhan Chen, Zhijie Lin, Ji Huang, Ling Yu, Lichuan Chen, Fan Chen, Xueli Yang, Zhennan Lin, Yansong Guo, Kaiyang Lin

**Affiliations:** a Department of Cardiology, Shengli Clinical Medical College of Fujian Medical University, Fujian Provincial Hospital, Fuzhou University Affiliated Provincial Hospital; b Fujian Provincial Key Laboratory of Cardiovascular Disease, Fujian Provincial Center for Geriatrics, Fujian Provincial Clinical Research Center for Severe Acute Cardiovascular Diseases, Fuzhou, China; c Center for Cardiovascular Epidemiology Research and Prevention of Fujian Provincial Hospital, Fuzhou, China; d Fujian Provincial Key Laboratory of Medical Big Data Engineering; e Tianjin Key Laboratory of Environment, Nutrition and Public Health, Department of Occupational and Environmental Health, School of Public Health, Tianjin Medical University, Tianjin, China

**Keywords:** Metals, Myocardial infarction, Environmental risk score, The Global Registry of Acute Coronary Events risk score

## Abstract

**Background:**

Metals such as arsenic, cadmium, and lead have been increasingly recognized as emerging cardiovascular risk factors, yet it is not considered in current clinical risk stratification for patients after myocardial infarction (MI).

**Methods:**

This prospective study consecutively enrolled 1,283 post-MI patients undergoing percutaneous coronary intervention between 2017 and 2021, with follow-up through April 2024. Baseline plasma levels of 19 metals were measured by inductively coupled plasma mass spectrometry. Associations between metals and all-cause mortality were evaluated using multivariable Cox proportional hazards models. Models were adjusted for age, sex, body mass index, smoking status, diabetes, hypertension, hyperlipidemia, estimated glomerular filtration rate, peak troponin I, prior PCI, and stent implantation. To assess joint associations of multiple metals, we fitted elastic net regularized Cox models and derived environmental metal risk scores (ERSs), which were added to the GRACE risk score to assess incremental prognostic value via C-statistics, time-dependent receiver operating characteristic curves, and reclassification metrics.

**Results:**

Among 1,283 patients, 122 all-cause mortality occurred during a median follow-up of 4.28 years. Higher plasma concentrations of copper, lead, and strontium were significantly associated with increased risks of all-cause mortality. Fully adjusted hazard ratios with 95% confidence intervals (per 1–standard deviation increase in log-transformed concentrations) were 1.67 (1.34–2.07) for copper, 1.26 (1.06–1.50) for lead, and 1.43 (1.20–1.71) for strontium. Higher ERS was also positively associated with all-cause mortality. Incorporation of ERS into the GRACE score improved discrimination at 5 years, increasing the area under the curve from 0.77 to 0.81 (*P* = 0.013), and significantly enhanced risk reclassification.

**Conclusions:**

Plasma copper, lead, and strontium levels were associated with all-cause mortality after MI, and further validation in independent cohorts is warranted before clinical implementation.

## Graphical abstract

**Figure:**
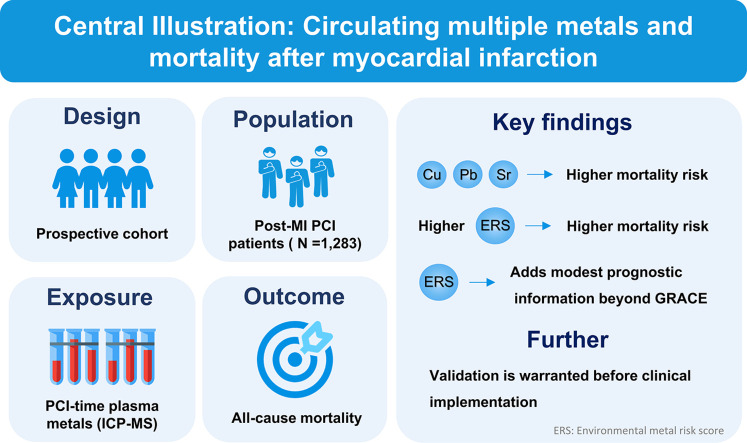

## 1 Introduction

Myocardial infarction (MI) is associated with a substantial risk of mortality [1,2]. Accurate risk stratification is essential for identifying high-risk patients, guiding clinical decision-making, and improving prognostic assessment. The Global Registry of Acute Coronary Events (GRACE) risk score remains one of the most widely adopted tools for risk stratification in patients after MI [3]. In external calibration analyses, Liu, Lüscher, et al. reported that GRACE 2.0 may be miscalibrated, with predicted mortality risk consistently lower than observed in real-world MI cohorts [4,5]. Previous studies have examined the incremental value of adding biomarkers such as C-reactive protein [6], NT-proBNP [7], Apo A-I, and KIM-1^8^to the GRACE score, with modest improvements in predictive performance. However, relatively little attention has been paid to exogenous environmental exposures, despite growing evidence suggesting the potential relevance of metals to mortality [9].

Environmental metal exposure has become prevalent in daily life, extending beyond traditional industrial settings [10]. Studies have linked exposure to metals such as arsenic, cadmium, and lead with adverse cardiovascular effects, including atherosclerosis, ischemic heart disease, and other cardiovascular disease [[11], [12], [13]]. However, most evidence to date has been derived from general populations, leaving uncertainty about whether metal exposure carries prognostic information in patients after MI and whether it can refine established clinical risk stratification. We therefore aimed to (i) quantify the associations of individual plasma metals with all-cause mortality after MI and (ii) develop environmental metal risk scores (ERSs) and test their incremental prognostic value when added to the GRACE score.

## 2 Materials and Methods

### 2.1 Study population

In this prospective cohort study conducted at Fujian Provincial Hospital from January 2017 to December 2021, we recruited 1344 consecutive patients diagnosed with MI and treated with percutaneous coronary intervention (PCI). The diagnosis of MI was made according to the Fourth Universal Definition of Myocardial Infarction, based on a combination of ischemic symptoms, electrocardiographic changes, dynamic elevations of cardiac troponin levels, and angiographic findings.

Patients were eligible for inclusion if they: (1) were aged 18 years or older; (2) had a confirmed diagnosis of MI and underwent PCI; (3) met health screening criteria (no congenital or valvular heart disease or other severe comorbidities and no uncontrolled psychiatric disorders); and (4) had no history of heavy metal poisoning or long-term occupational exposure to metals (e.g., welding, smelting, electroplating), as assessed at enrollment using a standardized questionnaire capturing prior physician-diagnosed metal poisoning and occupational history (industry, job title, and duration). We further excluded participants without available plasma samples (n=30) and those who died during hospitalization (n=31), resulting in a final analytic sample of 1283 participants. The study protocol was approved by the Ethics Committee of Fujian Provincial Hospital (approval number: K2017-01-012), and written informed consent was obtained from all participants prior to enrollment. A certificate of approval is available upon request.

### 2.2 Follow-up and definition of primary endpoint

This cohort was established specifically for the purpose of investigating environmental risk factors in post-MI prognosis, with predefined follow-up protocols. Trained research assistants conducted telephone interviews at 3, 6, 9, and 12 months after discharge, and annually thereafter, to collect information on recovery status, rehospitalizations, outpatient diagnoses, and deaths. Self-reported events were verified by reviewing available medical records, particularly for participants who continued care at the study site. All events were independently adjudicated by two cardiovascular specialists, with discrepancies resolved by a third expert. The adjudicators were blinded to exposure information throughout the review process.

Although follow-up of clinical outcomes is ongoing, the present analysis included data accrued through April 2024, with all-cause mortality selected as the primary outcome. Mortality was ascertained through multiple sources, including hospital medical records, reports from family members during follow-up, and the national death surveillance system maintained by the Fujian Provincial Center for Disease Control and Prevention. The date of death was defined as the date recorded in the official death registry or hospital documentation.

### 2.3 Analysis of plasma metals

Arterial blood samples were collected from participants through the radial artery at the beginning of PCI. Plasma was centrifuged at 4 °C, 3000 rpm for 5 minutes and stored at −80 °C in a freezer. The plasma concentrations of 25 metals, Aluminum (Al), As, Boron (B), Barium (Ba), Calcium (Ca), Cd, Cobalt (Co), Chromium (Cr), Cu, Iron (Fe), Potassium (K), Lithium (Li), Magnesium (Mg), Manganese (Mn), Mo, Sodium (Na), Nickel (Ni), Pb, Rubidium (Rb), Antimony (Sb), Se, Tin (Sn), Strontium (Sr), V, and Zinc (Zn), were measured using the NexION 350D inductively coupled plasma mass spectrometer (PerkinElmer, USA) in June 2023. The method for element detection was largely based on the protocol described in a previous study [14]. The limits of detection (LODs) and detection rates for plasma metals are presented in Table S1.

### 2.4 Data collection and variables

A range of variables were collected by trained interviewers at admission or extracted from medical records, including age (years), sex (male or female), body mass index (BMI, kg/m²), heart rate (beats per minute), systolic blood pressure (SBP, mmHg), smoking status (never or ever smoker), diabetes (yes or no), hypertension (yes or no), hyperlipidemia (yes or no), atrial fibrillation (yes or no), prior MI (yes or no), prior stroke (yes or no), prior PCI (yes or no), number of stents, ST-segment elevation myocardial infarction (STEMI, yes or no), Killip class (I, II, III, or IV), cardiac arrest at presentation (yes or no), electrocardiographic ischemia (yes or no), lipid profile (total cholesterol, triglycerides, low-density lipoprotein cholesterol, and high-density lipoprotein cholesterol; mmol/L), serum creatinine (μmol/L), estimated glomerular filtration rate (eGFR, mL/min/1.73 m²), cardiac troponin levels above the 99th percentile (yes or no), peak cardiac troponin I (cTnI; ng/mL), hemoglobin (HGB; g/L), and use of ticagrelor, aspirin, heparin, beta-blockers, statins, and angiotensin-converting enzyme inhibitors or angiotensin II receptor blockers (ACEi/ARBs; yes or no).

### 2.5 Risk score

In this study, the GRACE risk score was calculated at baseline by strictly adhering to the original variables and definitions derived from the GRACE registry and its key publications [3,15]. The score comprises eight components: age, heart rate, systolic blood pressure, serum creatinine, Killip class, ST-segment deviation on electrocardiography, cardiac arrest at presentation, and elevated cardiac biomarkers. It was used as the reference clinical risk model, against which the incremental prognostic value of metal exposure was evaluated.

### 2.6 Statistical analyses

Baseline characteristics were reported for all participants. Continuous variables are presented as mean [standard deviation (SD)] or median [interquartile range (IQR)], and categorical variables as frequency (percentage). After excluding metals with detection rates below 80% (Cd and Co) and macroelements (K, Ca, Na, and Mg), 19 plasma metals were selected for analysis. Spearman’s rank correlation was used to assess associations among these metals in 1,283 participants. Metal concentrations were log-transformed to reduce skewness in subsequent analyses.

Associations between plasma metal levels and the risks of all-cause mortality were assessed using Cox proportional hazards models, with hazard ratios (HRs) and 95% confidence intervals (CIs) reported. Two models were used for covariate adjustment. Model 1 included age, sex, body mass index (BMI), smoking status, diabetes, hypertension, hyperlipidemia, and estimated glomerular filtration rate (eGFR). Model 2 further adjusted for peak cardiac troponin I, prior PCI, and stent implantation. The proportional hazards assumption was assessed using Schoenfeld residuals, with no violations observed. Missing covariate data (including BMI and eGFR, both < 9%) were handled using multiple imputation. To account for multiple comparisons across 19 metals, *P*-values were adjusted using the Benjamini–Hochberg false discovery rate (FDR) method. Metals were first categorized into tertiles, and linear trends were assessed by modeling the median value of each tertile as a continuous variable. Additionally, restricted cubic spline models with four knots were used to flexibly assess non-linear associations.

To evaluate the potential joint effects of correlated metal exposures and address multicollinearity, we employed a two-stage analytical strategy. First, an elastic net regularized Cox proportional hazards model was fitted, including all 19 plasma metals as exposure variables, with covariates forced into the model to control for confounding. By combining the properties of the least absolute shrinkage and selection operator (LASSO) and ridge penalties, this approach can accommodate highly correlated exposures and mitigate multicollinearity while performing variable selection. The regularization parameter (lambda) was selected via 10-fold cross-validation to minimize mean squared error. Second, ERSs were constructed using the non-zero coefficients from the elastic net model to summarize the joint metal burden. ERSs provide a comprehensive summary indicator of exposure-related health risk in epidemiological studies. Two ERSs were generated: ERS1 comprised only metals that were statistically significant in the regular Cox model, weighted by their elastic net coefficients; ERS2 included all metals retained in the elastic net model, similarly weighted. ERS values were categorized into tertiles and included in Cox models to examine their associations with all-cause mortality. Proportional hazards assumptions were evaluated using Schoenfeld residuals.

To evaluate whether the ERS, as a composite metric of environmental exposure, provides incremental prognostic value beyond established clinical risk stratification, we conducted an exploratory analysis using the GRACE risk score as the reference model. Model discrimination was assessed by comparing Harrell’s C-statistic and time-dependent receiver operating characteristic (ROC) curves between the GRACE model and the GRACE-plus-ERS model. The incremental predictive performance associated with the addition of ERS was further quantified using integrated discrimination improvement (IDI) and category-based net reclassification improvement (categorical NRI). Categorical NRI was calculated using prespecified GRACE mortality risk categories (<3% low, 3–8% intermediate, ≥8% high) at 3 and 5 years [4]; continuous NRI was also reported. Calibration was assessed by plotting observed versus predicted risk across deciles, with observed risk estimated by the Kaplan–Meier method and calibration slope calculated by regressing observed on predicted risk. Clinical utility was evaluated using decision curve analysis (DCA) to compare net benefit across threshold probabilities. In a supplementary exploratory analysis, we also evaluated the change in the AUC after adding individual metals to the GRACE score.

Several sensitivity analyses were conducted to test the robustness of our findings. First, we excluded participants with a history of PCI. Second, the analyses were confined to participants without a history of MI or stroke at baseline. Third, we restricted the analyses to those aged ≤80 years or with an eGFR ≥60 mL/min/1.73 m² at baseline. All analyses were conducted using R software (version 4.2.0). Statistical significance was defined as a two-sided *P*-value <0.05.

## 3 Results

### 3.1 Participant characteristics

Table 1 describes the baseline characteristics of all participants. Of the 1283 participants, the mean age was 62.9 (±12.1) years, and 81.4% were male. We observed 122 all-cause mortality during a median follow-up of 4.28 years. Compared with survivors, participants who died were generally older and more likely to have comorbidities such as diabetes, hypertension, and atrial fibrillation. They also had a higher prevalence of prior MI, stroke, and PCI. They also exhibited clinical features associated with higher GRACE scores, such as elevated heart rate, lower systolic blood pressure, and a greater proportion with advanced Killip class.

**Table 1:** Baseline characteristics of all participants, and subgroups with MACEs and all-cause mortality.

	Participant group		
Characteristic	Total (n=1283)	No All-Cause Mortality (n = 1161)	All-Cause Mortality (n = 122)
Age, y	62.9 (12.1)	61.9 (11.9)	72.1 (10.0)
Male, n (%)	1045 (81.4)	953 (82.1)	92 (75.4)
BMI (kg/m^2^)	24.1 (3.17)	24.2 (3.13)	23.2 (3.37)
Heart rate (b.p.m.)	77.5 (13.3)	77.3 (13.1)	80.3 (15.4)
SBP (mmHg)	129 (115-143)	130 (115-143)	126(114;147)
Smoking status(yes), n (%)	657 (51.2)	601 (51.8)	56 (45.9)
Diabetes, n (%)	527 (41.1)	461 (39.7)	66 (54.1)
Hypertension, n (%)	786 (61.3)	698 (60.1)	88 (72.1)
Hyperlipidemia, n (%)	856 (66.7)	779 (67.1)	77 (63.1)
Atrial fibrillation, n (%)	76 (5.92)	58 (5.00)	18 (14.8)
Prior MI, n (%)	50 (3.90)	44 (3.79)	6 (4.92)
Prior stroke, n (%)	70 (5.46)	58 (5.00)	12 (9.84)
Prior PCI, n (%)	86 (6.70)	71 (6.12)	15 (12.3)
Number of stents	1.66 (0.95)	1.67 (0.95)	1.64 (0.94)
STEMI (%)	645 (50.3)	591 (50.9)	54 (44.3)
Killip class (%)			
I	811 (63.2)	763 (65.7)	48 (39.3)
II	337 (26.3)	295 (25.4)	42 (34.4)
III	54 (4.21)	38 (3.27)	16 (13.1)
IV	81 (6.31)	65 (5.60)	16 (13.1)
Cardiac arrest (%)	6 (0.47)	4 (0.34)	2 (1.64)
ECG ischaemia (%)	1034 (80.6)	932 (80.3)	102 (83.6)
Cholesterol, mmol/L	4.60 (1.27)	4.62 (1.26)	4.45 (1.40)
Triglyceride, mmol/L	1.52 (1.11-2.17)	1.53 (1.12-2.18)	1.35 (1.06-2.01)
LDL-C, mmol/L	3.08 (1.12)	3.09 (1.11)	2.95 (1.21)
HDL-C, mmol/L	0.99 (0.26)	0.98 (0.25)	1.02 (0.28)
Creatinine, μmol/L	89.6 (62.8)	85.5 (53.4)	129 (113)
eGFR, mL/min/1.73m^2^	109 (38.7)	111 (37.3)	82.3 (42.2)
Troponin >99th centile (%)	1225 (95.5)	1107 (95.3)	118 (96.7)
peak cTnI, ng/ml	19.5 (3.44-97.2)	19.5 (3.31-97.4)	21.1 (4.44-95.9)
Hemoglobin, g/L	137 (18.2)	139 (17.0)	123 (21.9)
Ticagrelor, n (%)	1007 (78.5)	923 (79.5)	84 (68.9)
Aspirin, n (%)	1278 (99.6)	1156 (99.6)	122 (100)
Heparin, n (%)	1276 (99.5)	1155 (99.5)	121 (99.2)
Beta-blocker, n (%)	1102 (85.9)	998 (86.0)	104 (85.2)
Statins, n (%)	1278 (99.6)	1156 (99.6)	122 (100)
ACEi/ARB, n (%)	1019 (79.4)	925 (79.7)	94 (77.0)

Values are mean ± SD, median (IQR) or n (%).

BMI, body mass index; SBP, systolic blood pressure; MI, myocardial infarction; PCI, percutaneous coronary intervention; STEMI, ST-segment elevation myocardial infarction; LDL-C, low density lipoprotein cholesterol; HDL-C, high density lipoprotein cholesterol; eGFR, estimated glomerular filtration rate; cTnI, cardiac troponin I; ACEi, angiotensin-converting enzyme inhibitor; ARB, angiotensin II receptor blocker.

### 3.2 Plasma metal levels and the risks of all-cause mortality

Supplementary Table S1 presents the distribution of plasma metal concentrations. The median (IQR) concentrations were 1126.09 µg/L (963.04–1355.82) for Cu, 20.23 µg/L (14.75–41.32) for Pb, and 109.40 µg/L (82.64–145.96) for Sr. Correlations between the plasma metals were predominantly positive and significant, as shown in Fig. S1.

After multivariable adjustment [Table 2 (Model 1)], higher plasma concentrations of copper, lead, and strontium were consistently and independently associated with an increased risk of all-cause mortality. Specifically, each 1–SD increase in log-transformed plasma copper, lead, and strontium concentrations was associated with a 67%, 26%, and 44% higher risk of all-cause mortality, respectively. These associations remained robust in the fully adjusted Model 2, whereas the associations for aluminum and nickel were attenuated to borderline significance. To enhance clinical interpretability, we additionally categorized key metals into tertiles. Consistent with the per–1 SD estimates, participants in the highest tertile had a higher mortality risk than those in the lowest tertile. In the fully adjusted model, the HRs for all-cause mortality comparing tertile 3 vs tertile 1 were 2.33 for copper, 1.77 for strontium, and 1.82 for lead (Table 3). Absolute event counts also increased across tertiles (deaths/N: copper 23/428 in tertile 1 vs 61/428 in tertile 3; lead 32/428 vs 55/428; strontium 31/428 vs 52/428). In restricted cubic spline analyses (Fig. S2), copper, lead, and strontium showed generally linear associations with all-cause mortality, with significant overall associations.

**Table 2:** Associations between plasma metal levels and the risks of all-cause mortality.

metals	Model 1*		Model 2†	
	HR (95% CIs)	*P*-FDR	HR (95% CIs)	*P*-FDR
ln Al	1.23(1.04-1.45)	0.044	1.22(1.03-1.45)	0.053
ln As	1.15(0.96-1.37)	0.215	1.13(0.94-1.35)	0.297
ln B	1.12(0.92-1.35)	0.335	1.11(0.91-1.34)	0.390
ln Ba	1.06(0.88-1.29)	0.681	1.06(0.87-1.28)	0.686
ln Cr	1.17(0.99-1.40)	0.145	1.17(0.98-1.39)	0.171
ln Cu	1.67(1.34-2.07)	<0.001	1.67(1.34-2.07)	<0.001
ln Fe	0.96(0.81-1.13)	0.706	0.95(0.80-1.12)	0.686
ln Li	1.12(0.93-1.34)	0.286	1.12(0.93-1.34)	0.297
ln Mn	1.22(1.02-1.46)	0.092	1.21(1.01-1.45)	0.110
ln Mo	1.18(0.98-1.43)	0.145	1.18(0.98-1.43)	0.155
ln Ni	1.25(1.05-1.48)	0.044	1.23(1.03-1.47)	0.063
ln Pb	1.26(1.06-1.50)	0.037	1.26(1.06-1.50)	0.041
ln Rb	1.20(0.99-1.45)	0.111	1.19(0.99-1.44)	0.141
ln Sb	1.08(0.91-1.28)	0.558	1.08(0.91-1.28)	0.549
ln Se	1.00(0.83-1.19)	0.955	0.97(0.81-1.17)	0.788
ln Sn	0.98(0.82-1.17)	0.828	0.98(0.82-1.17)	0.788
ln Sr	1.44(1.22-1.71)	<0.001	1.43(1.20-1.71)	<0.001
ln V	1.26(1.06-1.50)	0.037	1.24(1.04-1.48)	0.053
ln Zn	1.02(0.86-1.22)	0.797	1.02(0.86-1.22)	0.788

Bold indicates statistically significant differences (*P*<0.05).

⁎ Model 1 was adjusted by age, sex, BMI, smoking status diabetes, hypertension, hyperlipidemia, eGFR.

† Model 2 was further adjusted for peak cardiac troponin I, history of prior PCI, and stent implantation. HRs were estimated per 1–SD increase in log-transformed plasma metal concentrations.

**Table 3:** Hazard ratio (95% CIs) of all-cause mortality by baseline plasma metal levels.

Subgroup	Deaths / N	Model 1*	Model 2†
ln Cu			
Tertile 1	23/428	Ref.	Ref.
Tertile 2	38/427	1.48(0.88-2.49)	1.53(0.91-2.58)
Tertile 3	61/428	2.29(1.41-3.73)	2.33(1.43-3.80)
*P*-Trend		<0.001	<0.001
ln Sr			
Tertile 1	31/428	Ref.	Ref.
Tertile 2	39/427	1.34(0.82-2.17)	1.39(0.85-2.27)
Tertile 3	52/428	1.75(1.12-2.74)	1.77(1.13-2.78)
*P*-Trend		0.012	0.007
ln Pb			
Tertile 1	32/428	Ref.	Ref.
Tertile 2	35/427	1.28(0.79-2.06)	1.30(0.80-2.09)
Tertile 3	55/428	1.88(1.18-3.00)	1.82(1.13-2.92)
*P*-Trend		0.017	0.017

Bold indicates statistically significant differences (*P*<0.05).

⁎ Model 1 was adjusted by age, sex, BMI, smoking status, diabetes, hypertension, hyperlipidemia, eGFR.

† Model 2 was further adjusted for peak cardiac troponin I, history of prior PCI, and stent implantation.

### 3.3 Multi-metal analyses

The results of metal selection from the Cox elastic net models are shown in Table S2. Of the 19 candidate metals, 11 (As, Ba, Cu, Fe, Mo, Pb, Rb, Se, Sn, Sr, and Zn) were associated with all-cause mortality. Cu, Pb, and Sr, which were significant in the standard Cox model, were not only retained in the models but also ranked among the top three in terms of weights (1.126, 0.241, and 0.795, respectively) among the selected metals. Table 4 shows a positive association between ERS and the risk of all-cause mortality. In tertile-based analyses, compared with participants in the lowest tertile, those in the highest tertile of ERS1 had a significantly higher risk of all-cause mortality (HR: 2.33, 95% CI: 1.44–3.77; *P*-trend <0.001), and a stronger association was observed for ERS2 (HR: 3.63, 95% CI: 2.11–6.26; *P*-trend <0.001). When modeled continuously, the associations were more modest: per 1–SD increase, the fully adjusted HR was 1.72 (95% CI: 1.43–2.08) for ERS1 and 2.10 (95% CI: 1.73–2.54) for ERS2. Proportional hazards assumptions were evaluated using Schoenfeld residuals, with no evidence of violation for ERS tertiles (all *P* > 0.20) and non-significant global tests (all *P* > 0.25).

**Table 4:** Associations of ERS with all-cause mortality.

Subgroup	Deaths / N	Model 1*	Model 2†
ERS1^a^			
ERS1, per SD increase		1.72(1.43-2.06)	1.72(1.43-2.08)
ERS1-T1	24/428	Ref.	Ref.
ERS1-T2	37/427	1.64(0.98-2.76)	1.65(0.98-2.77)
ERS1-T3	61/428	2.39(1.48-3.85)	2.33(1.44-3.77)
*P*-Trend		<0.001	<0.001
ERS2^b^			
ERS2, per SD increase		2.08(1.73-2.50)	2.10(1.73-2.54)
ERS2-T1	16/428	Ref.	Ref.
ERS2-T2	21/427	1.14(0.59-2.19)	1.14(0.59-2.19)
ERS2-T3	85/428	3.60(2.09-6.20)	3.63(2.11-6.26)
*P*-Trend		<0.001	<0.001

Bold indicates statistically significant differences (*P*<0.05). ERS, environmental metal risk score;

a ERS1 includes metals significant in the Cox model, weighted by elastic net coefficients;

b ERS2 includes all metals retained by the elastic net model, similarly weighted;

⁎ Model 1 was adjusted by age, sex, BMI, smoking status, diabetes, hypertension, hyperlipidemia, eGFR.

† Model 2 was further adjusted for peak cardiac troponin I, history of prior PCI, and stent implantation.

### 3.4 Added predictive value of ERS over the GRACE model

**Fig. 1:**
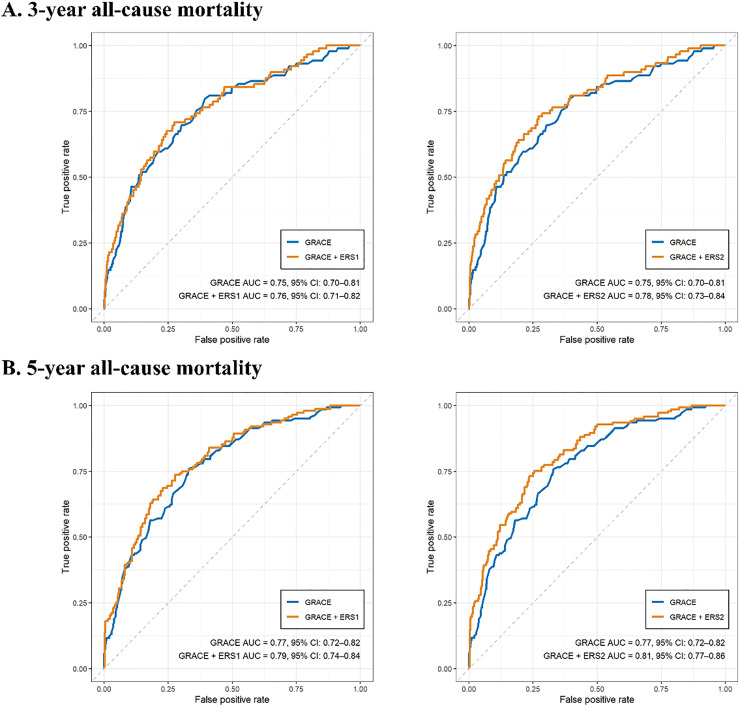
Time-dependent receiver operating characteristic (time ROC) curves assessing the predictive performance of GRACE, GRACE+ERS1, and GRACE+ERS2 for 3-year and 5-year all-cause mortality. GRACE, Global Registry of Acute Coronary Events risk score; ERS, environmental metal risk score; ERS1 includes metals significant in the Cox model, weighted by elastic net coefficients; ERS2 includes all metals retained by the elastic net model, similarly weighted; AUC, area under the curve.

As shown in Fig. 1, all models demonstrated modestly improved predictive performance over time, with higher AUCs observed at 5 years than at 3 years for all-cause mortality. At 3 years, the addition of ERS1 or ERS2 to the GRACE score resulted in small increases in AUC compared with GRACE alone. At 5 years, incorporation of ERS2 led to a statistically significant improvement in discrimination, with the AUC increasing from 0.77 to 0.81 (*P* = 0.013), whereas the improvement associated with ERS1 was more modest.

The addition of ERS was associated with improved risk reclassification and discrimination for all-cause mortality (Table 5). At 5 years, incorporation of ERS2 into the GRACE model yielded significant improvements in categorical NRI (0.21; 95% CI: 0.09–0.34) and IDI (0.07; 95% CI: 0.04–0.10). The C-statistic increased from 0.736 for GRACE to 0.780 with the inclusion of ERS2 (ΔC = 0.044; *P* < 0.05), indicating improved discrimination. By contrast, ERS1 provided smaller incremental improvements. Calibration plots and calibration slopes are presented in Fig. S3, and DCA curves at 3 and 5 years are shown in Fig. S4. Time-dependent ROC curves for the GRACE model with the addition of individual metals at 3 and 5 years are presented in Fig. S5.

**Table 5:** Added predictive ability and reclassification statistics of ERS.

	GRACE	GRACE+ERS1^a^	GRACE+ERS2^b^
Categorical NRI (95%Cl) at 3years		0.08(-0.05–0.19)	0.18(0.05–0.32)
NRI+ (95%Cl)		0.00(-0.10–0.08)	0.03(-0.08–0.12)
NRI- (95%Cl)		0.08(0.02–0.16)	0.15(0.09–0.25)
Continuous NRI (95%Cl) at 3years	Ref.	0.20(-0.04-0.49)	0.61(0.37-0.79)
IDI (95% CI) at 3years	Ref.	0.03(0.01-0.06)	0.06(0.03-0.09)
Categorical NRI (95%Cl) at 5 years		0.08(-0.01–0.17)	0.21(0.09–0.34)
NRI+ (95%Cl)		-0.01(-0.07–0.06)	0.00(-0.07–0.09)
NRI- (95%Cl)		0.09(0.01–0.15)	0.21(0.12–0.31)
Continuous NRI (95%Cl) at 5 years	Ref.	0.29(0.09-0.58)	0.69(0.47-0.90)
IDI (95% CI) at 5 years	Ref.	0.03(0.01-0.07)	0.07(0.04-0.10)
C-statistic	0.736	0.751	0.780
ΔC-statistic	Ref.	0.015	0.044 c

CI, confidence interval; IDI, integrated discrimination improvement; NRI, net reclassification improvement; GRACE, Global Registry of Acute Coronary Events risk score; ERS, environmental metal risk score;

a ERS1 includes metals significant in the Cox model, weighted by elastic net coefficients;

b ERS2 includes all metals retained by the elastic net model, similarly weighted;

c C-statistics were statistically significantly improved (*P* < 0.05); Categorical NRI used prespecified GRACE mortality risk categories (<3%, 3–8%, ≥8%) at 3 and 5 years.

### 3.5 Sensitivity analyses

In sensitivity analyses, the associations between plasma metal levels and the risks of all-cause mortality remained largely unchanged. Similar results were observed after excluding participants with a history of PCI (Table S3), MI (Table S4), or stroke (Table S5) at baseline. The associations also remained stable when analyses were restricted to participants aged ≤80 years (Table S6) or those with eGFR ≥60 mL/min/1.73 m² at baseline (Table S7).

## 4 Discussion

In this prospective cohort study, we evaluated the associations between plasma concentrations of individual and multiple metals and the risk of all-cause mortality among patients after MI. Higher plasma levels of copper, lead, and strontium were significantly associated with an increased risk of all-cause mortality. Using a Cox elastic net model, copper, lead, and strontium were identified as the primary metal predictors of mortality. Furthermore, the ERS, a composite metric reflecting combined metal exposure, was positively associated with all-cause mortality. Importantly, incorporation of ERS into the GRACE risk model provided modest incremental prognostic information. These findings provide new epidemiological evidence on the prognostic relevance of metal exposure in patients after MI and underscore the potential value of integrating ERS into clinical risk stratification.

Our findings indicate that elevated plasma copper levels were positively associated with all-cause mortality in patients. Although data on copper exposure in clinical populations are scarce, our findings align with prior prospective cohort studies. For instance, the MESA cohort reported that higher copper levels were associated with increased risks of all-cause mortality [16]. Similarly, the DF-TJ cohort found that individuals in the highest quartile of plasma copper had a 73% and 94% higher risk of all-cause and cardiovascular mortality, respectively [17]. Furthermore, a meta-analysis including over 348,000 participants also linked elevated serum copper exposure to increased cardiovascular disease risk [18]. The latest research suggests that copper exhibits biphasic effects—being essential at physiological levels but potentially harmful when in excess [19]. However, epidemiological studies, including ours, have often reported an approximately linear association [16]. This pattern may reflect not only external exposure but also disease-related perturbations in copper homeostasis (e.g., inflammation or oxidative stress), raising the possibility that circulating copper serves as both an exposure marker and a marker of disease severity [20]. Given the observational design, it remains unclear whether copper is a causal factor or a marker of disease. Further mechanistic and Mendelian randomization studies are needed to clarify its impact.

Lead is a persistent and bioaccumulative toxicant, and no established safe level of exposure has been identified [21,22]. Although previous studies have adequately demonstrated the significant cardiovascular toxicity of lead [23], most of these studies were conducted in healthy populations [18]. In our analysis, higher plasma lead levels were significantly associated with an increased risk of all-cause mortality. Heart failure is a major contributor to mortality in patients after MI [24,25]. Lead exposure has been shown to impair endothelial nitric oxide synthase activity, leading to endothelial dysfunction, increased peripheral vascular resistance, and elevated blood pressure [26]. These vascular alterations increase left ventricular afterload and oxidative stress, thereby exacerbating cardiac dysfunction and potentially contributing to higher mortality [27].

To our knowledge, this is among the first studies to systematically evaluate the prognostic relevance of plasma strontium in patients after MI. Patients in the highest tertile had significantly higher risks of all-cause mortality compared with those in the lowest tertile. Most previous research has focused on strontium’s role in bone metabolism [28], while its cardiovascular effects remain controversial. A population-based study in 24 Texas communities found an inverse association between strontium concentrations in drinking water and cardiovascular mortality in adults [29]. Nonetheless, other studies have linked strontium exposure to increased cardiovascular risk. For example, plasma strontium levels have been associated with elevated cardiovascular risk in patients with type 2 diabetes [30]. The European Medicines Agency has also recommended restricting the use of strontium ranelate in the treatment of osteoporosis due to an elevated risk of MI [31]. These conflicting findings may reflect differences in exposure levels or individual metabolic variation. Further research is needed to clarify the underlying mechanisms and clinical implications.

In real-world settings, individuals are exposed to multiple metals simultaneously rather than single elements, making it important to assess their combined effects [32]. Using a Cox elastic-net model incorporating 19 plasma metals, we identified copper, lead, and strontium as the strongest predictors of all-cause mortality. The GRACE score remains one of the most widely adopted tools for risk stratification in patients after MI, relying on clinical characteristics and routine laboratory markers [3]. Previous efforts to enhance its predictive performance have focused on circulating biomarkers such as C-reactive protein, NT-proBNP, Apo A-I, and KIM-1, as well as algorithmic updates [[5], [6], [7], [8]]. In our study, adding a metal-based ERS to the GRACE score yielded a modest improvement in long-term mortality discrimination, suggesting that cumulative metal burden may provide incremental prognostic information beyond traditional GRACE components. We further evaluated calibration and clinical utility using calibration plots and DCA. Calibration slopes were close to 1 at both 3 and 5 years, with modest improvement after adding ERS at 3 years but less evident change at 5 years. DCA suggested net benefit for ERS-augmented models at low-to-moderate threshold probabilities, which attenuated at higher thresholds. Collectively, these findings suggest that ERS may offer incremental value as an adjunct to GRACE, but the extent of clinical impact warrants further validation. While plasma metal measurement currently requires specialized laboratory infrastructure and incurs additional costs, the ongoing advancement of high-throughput, multiplexed assays is expected to improve feasibility and reduce expense over time. Future studies should further evaluate the cost-effectiveness, implementation feasibility, and potential health benefits of incorporating ERS into routine care.

## 5 Limitations

First, metals were assessed at a single baseline time point during the index hospitalization, which may not reliably capture chronic exposure patterns or post-discharge trajectories. Second, exposure misclassification cannot be fully excluded, because occupational exposure and prior heavy metal poisoning were ascertained by questionnaire/case report form rather than quantitative exposure assessment. Third, reverse causation cannot be fully excluded. Plasma metal concentrations measured during acute MI may reflect not only longer-term environmental exposure but also acute pathophysiological changes and in-hospital management (e.g., inflammation, organ dysfunction, nutritional status, intravenous fluids, or medications). Fourth, residual confounding is possible. Kidney function is a key determinant of metal clearance; although we adjusted for eGFR, confounding related to renal handling and other unmeasured factors cannot be fully excluded. Fifth, the ERS was derived using an elastic net regularized Cox model and evaluated in the same cohort. Although cross-validation was used, overfitting and optimistic estimates of discrimination improvement cannot be excluded. Therefore, the incremental prognostic performance should be interpreted cautiously and requires external validation in independent cohorts.

## 6 Conclusions

In this prospective cohort of patients after MI, higher plasma concentrations of copper, lead, and strontium were independently associated with an increased risk of all-cause mortality. A multi-metal ERS was also associated with mortality and provided statistically significant, albeit modest, incremental prognostic information beyond the GRACE risk model. These observational findings require further validation in independent cohorts before any consideration of clinical implementation.

## Ethics statement

The study protocol was approved by the Ethics Committee of Fujian Provincial Hospital (approval number: K2017-01-012), and written informed consent was obtained from all participants prior to enrollment. A certificate of approval is available upon request.

## Funding

This work was sponsored by the 10.13039/501100014125Fujian Provincial Health Technology Project (Grant No. 2022QNA002); the 10.13039/100003536Youth Top Talent Project of Fujian Provincial Foal Eagle Program; Fujian Research and Training Grants for Young and Middle-aged Leaders in Health Care; the Joint Funds for the Innovation of Science and Technology, Fujian Province (Grant No. 2024Y9071); the 10.13039/100012427Joint Funds for the Innovation of Science and Technology, Fujian Province (Grant No. 2023Y9321) and the 10.13039/501100012603Startup Fund for Scientific Research, Fujian Medical University (Grant No. 2022QH1305).

## Author Statement

This manuscript has not been published previously in any form and is not under consideration elsewhere. All authors and relevant institutional authorities have approved its submission. If accepted, it will not be published elsewhere in the same form, in any language, without the copyright holder’s written consent.

## CRediT authorship contribution statement

**Minmin Wu:** Writing – original draft, Methodology, Formal analysis, Conceptualization. **Junhan Chen:** Visualization, Software. **Zhijie Lin:** Formal analysis, Data curation. **Ji Huang:** Investigation, Data curation. **Ling Yu:** Investigation. **Lichuan Chen:** Investigation. **Fan Chen:** Investigation. **Xueli Yang:** Writing – review & editing. **Zhennan Lin:** Writing – review & editing. **Yansong Guo:** Supervision, Project administration, Funding acquisition. **Kaiyang Lin:** Supervision, Resources, Project administration.

## Declaration of competing interest

The authors declare that they have no known competing financial interests or personal relationships that could have appeared to influence the work reported in this paper.

## Appendix Supplementary materials

Image, application 1

## References

[1] Song J., Murugiah K., Hu S., Gao Y., Li X., Krumholz H. (2020). Incidence, predictors, and prognostic impact of recurrent acute myocardial infarction in china. Heart.

[2] Van de Werf F., Bax J., Betriu A., Blomstrom-Lundqvist C., Crea F., Falk V. (2008). Management of acute myocardial infarction in patients presenting with persistent st-segment elevation: the task force on the management of st-segment elevation acute myocardial infarction of the european society of cardiology. Eur Heart J.

[3] Fox K., Dabbous O., Goldberg R., Pieper K., Eagle K., Van de Werf F. (2006). Prediction of risk of death and myocardial infarction in the six months after presentation with acute coronary syndrome: prospective multinational observational study (grace). Bmj.

[4] Hung J., Roos A., Kadesjö E., McAllister D., Kimenai D., Shah A. (2021). Performance of the grace 2.0 score in patients with type 1 and type 2 myocardial infarction. Eur Heart J.

[5] Wenzl F., Kraler S., Ambler G., Weston C., Herzog S., Räber L. (2022). Sex-specific evaluation and redevelopment of the grace score in non-st-segment elevation acute coronary syndromes in populations from the uk and switzerland: A multinational analysis with external cohort validation. Lancet.

[6] Schiele F., Meneveau N., Seronde M., Chopard R., Descotes-Genon V., Dutheil J. (2010). C-reactive protein improves risk prediction in patients with acute coronary syndromes. Eur Heart J.

[7] Khan S., Narayan H., Ng K., Dhillon O., Kelly D., Quinn P. (2009). N-terminal pro-b-type natriuretic peptide complements the grace risk score in predicting early and late mortality following acute coronary syndrome. Clin Sci (L).

[8] Toprak B., Weimann J., Lehmacher J., Haller P., Hartikainen T., Schock A. (2024). Prognostic utility of a multi-biomarker panel in patients with suspected myocardial infarction. Clin Res Cardiol.

[9] Lamas G., Bhatnagar A., Jones M., Mann K., Nasir K., Tellez-Plaza M. (2023). Contaminant metals as cardiovascular risk factors: a scientific statement from the american heart association. J Am Heart Assoc.

[10] (2012). Health risks from toxic pollution. Lancet.

[11] Barregard L., Sallsten G., Harari F., Andersson E., Forsgard N., Hjelmgren O. (2021). Cadmium exposure and coronary artery atherosclerosis: a cross-sectional population-based study of swedish middle-aged adults. Env Health Perspect.

[12] Jain N., Potula V., Schwartz J., Vokonas P., Sparrow D., Wright R. (2007). Lead levels and ischemic heart disease in a prospective study of middle-aged and elderly men: the va normative aging study. Env Health Perspect.

[13] Sears C., Eliot M., Raaschou-Nielsen O., Poulsen A., Harrington J., Howe C. (2022). Urinary cadmium and incident heart failure: a case-cohort analysis among never-smokers in denmark. Epidemiology.

[14] Yuan Y., Xiao Y., Feng W., Liu Y., Yu Y., Zhou L. (2017). Plasma metal concentrations and incident coronary heart disease in chinese adults: the dongfeng-tongji cohort. Env Health Perspect.

[15] Eagle K., Lim M., Dabbous O., Pieper K., Goldberg R., Van de Werf F. (2004). A validated prediction model for all forms of acute coronary syndrome: estimating the risk of 6-month postdischarge death in an international registry. Jama.

[16] Martinez-Morata I., Schilling K., Glabonjat R., Domingo-Relloso A., Mayer M., McGraw K. (2024). Association of urinary metals with cardiovascular disease incidence and all-cause mortality in the multi-ethnic study of atherosclerosis (mesa). Circulation.

[17] Shi L., Yuan Y., Xiao Y., Long P., Li W., Yu Y. (2021). Associations of plasma metal concentrations with the risks of all-cause and cardiovascular disease mortality in chinese adults. Env Int.

[18] Chowdhury R., Ramond A., O'Keeffe L., Shahzad S., Kunutsor S., Muka T. (2018). Environmental toxic metal contaminants and risk of cardiovascular disease: systematic review and meta-analysis. Bmj.

[19] Wang D., Tian Z., Zhang P., Zhen L., Meng Q., Sun B. (2023). The molecular mechanisms of cuproptosis and its relevance to cardiovascular disease. Biomed Pharmacother.

[20] Yang Y., Wu J., Wang L., Ji G., Dang Y. (2024). Copper homeostasis and cuproptosis in health and disease. MedComm (2020).

[21] Harari F., Barregard L., Östling G., Sallsten G., Hedblad B., Forsgard N. (2019). Blood lead levels and risk of atherosclerosis in the carotid artery: results from a swedish cohort. Env Health Perspect.

[22] Lanphear B., Rauch S., Auinger P., Allen R., Hornung R.W. (2018). Low-level lead exposure and mortality in us adults: a population-based cohort study. Lancet Public Health.

[23] Patwa J., Flora S.J.S. (2020). Heavy metal-induced cerebral small vessel disease: insights into molecular mechanisms and possible reversal strategies. Int J Mol Sci.

[24] Butler J., Hammonds K., Talha K., Alhamdow A., Bennett M., Bomar J. (2025). Incident heart failure and recurrent coronary events following acute myocardial infarction. Eur Heart J.

[25] Gerber Y., Weston S., Enriquez-Sarano M., Berardi C., Chamberlain A., Manemann S. (2016). Mortality associated with heart failure after myocardial infarction: a contemporary community perspective. Circ Heart Fail.

[26] Lamas G., Ujueta F., Navas-Acien A. (2021). Lead and cadmium as cardiovascular risk factors: the burden of proof has been met. J Am Heart Assoc.

[27] Jomova K., Valko M. (2011). Advances in metal-induced oxidative stress and human disease. Toxicology.

[28] Kołodziejska B., Stępień N., Kolmas J. (2021). The influence of strontium on bone tissue metabolism and its application in osteoporosis treatment. Int J Mol Sci.

[29] Dawson E., Frey M., Moore T., McGanity W.J. (1978). Relationship of metal metabolism to vascular disease mortality rates in texas. Am J Clin Nutr.

[30] Long T., Wang R., Wang J., Wang F., Xu Y., Wei Y. (2019). Plasma metals and cardiovascular disease in patients with type 2 diabetes. Env Int.

[31] Bolland M., Grey A. (2013). Strontium and cardiovascular events. Ann Rheum Dis.

[32] Guo X., Li N., Wang H., Su W., Song Q., Liang Q. (2022). Combined exposure to multiple metals on cardiovascular disease in nhanes under five statistical models. Env Res.

