# Supplementary material for: Circulating multiple metals and mortality after myocardial infarction: incremental value beyond GRACE
Source: Am J Prev Cardiol. 2026 Mar 15;27:101548. doi: 10.1016/j.ajpc.2026.101548 (PMC13261272; doi:10.1016/j.ajpc.2026.101548)
Supplement: Supplementary file 1 [file mmc1.docx]

| **Table S1. Concentrations of metals in plasma samples** | | | | | |
| --- | --- | --- | --- | --- | --- |
| **Metals** | **LOD** | **Detection rate (%)** | **25^th^** | **50^th^** | **75^th^** |
| Al (µg/L) | 0.9530 | 99.92% | 267.81 | 451.66 | 613.20 |
| As(µg/L) | 0.0270 | 99.85% | 3.19 | 5.63 | 9.25 |
| B(µg/L) | 1.3810 | 82.80% | 40.60 | 110.43 | 239.67 |
| Ba(µg/L) | 0.0220 | 100.00% | 74.69 | 108.10 | 442.82 |
| Ca(mg/L) | 0.0182 | 99.92% | 142.60 | 159.62 | 185.29 |
| Cd(µg/L) | 0.0130 | 18.42% | ＜LOD | ＜LOD | ＜LOD |
| Co(µg/L) | 0.0067 | 8.68% | ＜LOD | ＜LOD | ＜LOD |
| Cr(µg/L) | 0.0260 | 99.92% | 7.39 | 9.83 | 13.56 |
| Cu(µg/L) | 0.0370 | 99.92% | 963.04 | 1,126.09 | 1,355.82 |
| Fe(µg/L) | 1.0810 | 99.85% | 621.89 | 963.37 | 1,386.26 |
| K(mg/L) | 0.0164 | 100.00% | 793.00 | 947.80 | 1,189.88 |
| Li(µg/L) | 0.2540 | 98.25% | 40.39 | 50.46 | 58.13 |
| Mg(mg/L) | 0.0029 | 99.92% | 24.96 | 28.24 | 32.45 |
| Mn(µg/L) | 0.0150 | 99.85% | 6.67 | 12.25 | 17.69 |
| Mo(µg/L) | 0.0400 | 93.61% | 1.30 | 2.03 | 3.19 |
| Na(mg/L) | 0.0087 | 100.00% | 3,967.47 | 4,292.71 | 4,821.27 |
| Ni(µg/L) | 0.0450 | 99.92% | 4.88 | 6.82 | 10.36 |
| Pb(µg/L) | 0.0130 | 99.92% | 14.75 | 20.23 | 41.32 |
| Rb(µg/L) | 0.0480 | 99.92% | 318.38 | 433.81 | 608.40 |
| Sb(µg/L) | 0.0071 | 92.69% | 0.23 | 0.38 | 0.61 |
| Se(µg/L) | 0.4720 | 99.92% | 106.40 | 128.45 | 154.59 |
| Sn(µg/L) | 0.2420 | 99.92% | 16.09 | 22.85 | 33.49 |
| Sr(µg/L) | 0.0310 | 99.92% | 82.64 | 109.40 | 145.96 |
| V(µg/L) | 0.0060 | 100.00% | 4.80 | 5.65 | 6.61 |
| Zn(µg/L) | 0.7510 | 99.92% | 955.10 | 1,464.91 | 2,616.89 |
| <LOD denotes values below the limit of detection. | | | | | |

**
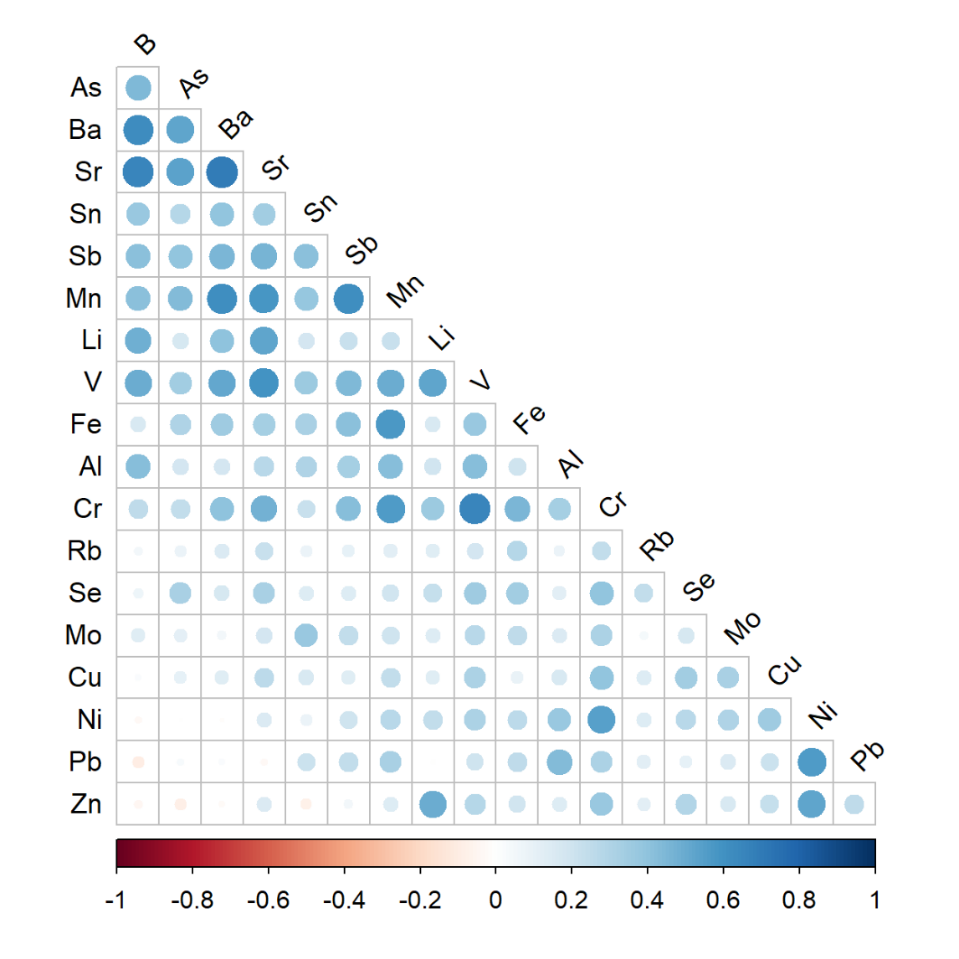
**

**Figure S1.** Spearman correlation matrix of plasma metals.


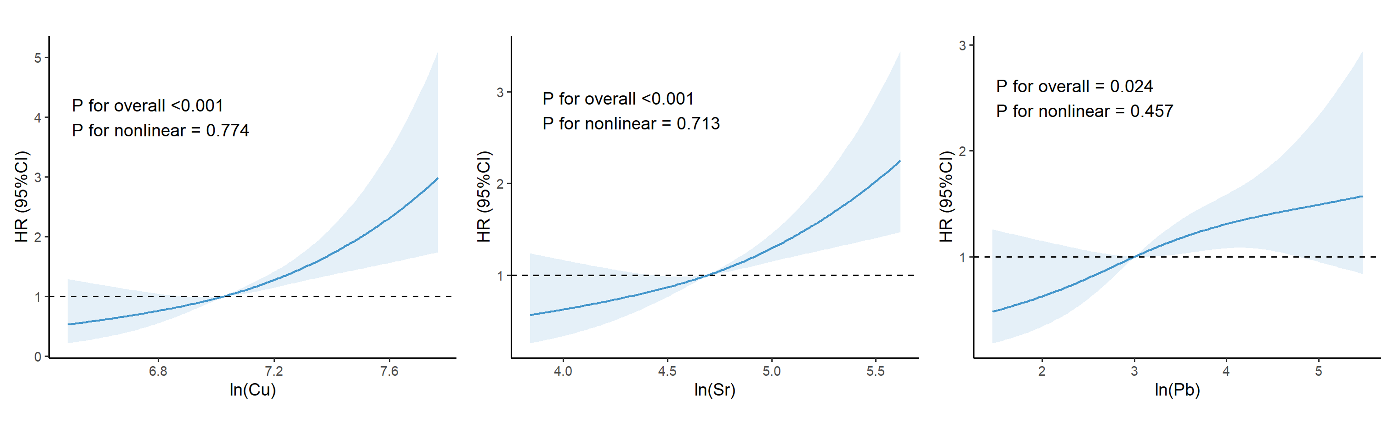


**Figure S2.** Restricted cubic spline plots showing the associations between metal exposure and all-cause mortality. The curves represent adjusted hazard ratios for the natural log-transformed concentrations of copper, strontium, and lead in the Cox regression model. The model was adjusted for age, sex, BMI, smoking status, diabetes, hypertension, hyperlipidemia, eGFR, peak cardiac troponin I, history of prior PCI, and stent implantation.

| **Table S2. Estimated environmental risk score (ERS) weights (regression coefficient) for All-Cause Mortality.** | |
| --- | --- |
| **Metal** | **Weights** |
| ln Al | . |
| ln As | 0.027 |
| ln B | . |
| ln Ba | -0.060 |
| ln Cr | . |
| **ln Cu** | **1.126** |
| ln Fe | -0.181 |
| ln Li | . |
| ln Mn | . |
| ln Mo | 0.134 |
| ln Ni | . |
| **ln Pb** | **0.241** |
| ln Rb | 0.100 |
| ln Sb | . |
| ln Se | -0.686 |
| ln Sn | -0.182 |
| **ln Sr** | **0.795** |
| ln V | . |
| ln Zn | -0.154 |
| Elastic Net model was adjusted by age, sex, BMI, smoking status, diabetes, hypertension, hyperlipidemia, eGFR, peak cardiac troponin I, history of prior PCI, and stent implantation. | |

| **Table S3. Hazard Ratios (95% CI) for All-Cause Mortality in participants without** **history of prior PCI at baseline.** | | |
| --- | --- | --- |
| **Subgroup** | **All-Cause Mortality(n=107)** | |
|  | **Cases/noncases** | **HR (95% CI)** |
| ln Cu |  | |
| Tertile 1 | 20/400 | Ref. |
| Tertile 2 | 34/399 | 1.55(0.88-2.71) |
| Tertile 3 | 53/398 | **2.31(1.36-3.90)** |
| *P*-Trend | <0.001 | |
| ln Sr |  | |
| Tertile 1 | 29/407 | Ref. |
| Tertile 2 | 34/405 | 1.23(0.74-2.03) |
| Tertile 3 | 44/385 | **1.94(1.18-3.18)** |
| *P*-Trend | 0.009 | |
| ln Pb |  | |
| Tertile 1 | 28/402 | Ref. |
| Tertile 2 | 31/402 | 1.27(0.76-2.13) |
| Tertile 3 | 48/393 | **1.65(1.02-2.68)** |
| *P*-Trend | 0.040 | |
| Bold indicates statistically significant differences (*P*<0.05).  Cox regression model was adjusted by age, sex, BMI, smoking status, diabetes, hypertension, hyperlipidemia, eGFR, peak cardiac troponin I, history of prior PCI, and stent implantation. | | |

| **Table S4. Hazard Ratios (95% CI) for All-Cause Mortality in participants** **without history of prior MI at baseline.** | | | |
| --- | --- | --- | --- |
| **Subgroup** | **All-Cause Mortality(n=116)** | | |
|  | **Cases/noncases** | | **HR (95% CI)** |
| ln Cu |  | | |
| Tertile 1 | 23/411 | | Ref. |
| Tertile 2 | 35/411 | | 1.46(0.86-2.50) |
| Tertile 3 | 58/411 | | **2.34(1.43-3.83)** |
| *P*-Trend | <0.001 | | |
| ln Sr |  | | |
| Tertile 1 | 29/416 | | Ref. |
| Tertile 2 | 38/409 | | 1.42(0.87-2.32) |
| Tertile 3 | 49/408 | | **2.05(1.27-3.33)** |
| *P*-Trend | 0.003 | | |
| ln Pb |  | | |
| Tertile 1 | 31/412 | | Ref. |
| Tertile 2 | 35/412 | | 1.34(0.82-2.19) |
| Tertile 3 | 50/409 | | **1.59(1.01-2.53)** |
| *P*-Trend |  |  | 0.062 |
| Bold indicates statistically significant differences (*P*<0.05).  Cox regression model was adjusted by age, sex, BMI, smoking status, diabetes, hypertension, hyperlipidemia, eGFR, peak cardiac troponin I, history of prior PCI, and stent implantation. | | | |

| **Table S5. Hazard Ratios (95% CI) for All-Cause Mortality in participants** **without history of prior stroke at baseline.** | | |
| --- | --- | --- |
| **Subgroup** | **All-Cause Mortality(n=110)** | |
|  | **Cases/noncases** | **HR (95% CI)** |
| ln Cu |  | |
| Tertile 1 | 21/408 | Ref. |
| Tertile 2 | 34/407 | 1.56(0.90-2.71) |
| Tertile 3 | 55/398 | **2.42(1.45-4.04)** |
| *P*-Trend | <0.001 | |
| ln Sr |  | |
| Tertile 1 | 29/404 | Ref. |
| Tertile 2 | 33/407 | 1.12(0.68-1.87) |
| Tertile 3 | 48/402 | **1.75(1.07-2.87)** |
| *P*-Trend | 0.018 | |
| ln Pb |  | |
| Tertile 1 | 28/406 | Ref. |
| Tertile 2 | 32/401 | 1.54(0.91-2.61) |
| Tertile 3 | 50/406 | **1.93(1.20-3.12)** |
| *P*-Trend | 0.012 | |
| Bold indicates statistically significant differences (*P*<0.05).  Cox regression model was adjusted by age, sex, BMI, smoking status, diabetes, hypertension, hyperlipidemia, eGFR, peak cardiac troponin I, history of prior PCI, and stent implantation. | | |

| **Table S6. Hazard Ratios (95% CI) for All-Cause Mortality in participants** **aged ≤80 years at baseline.** | | |
| --- | --- | --- |
| **Subgroup** | **All-Cause Mortality(n=95)** | |
|  | **Cases/noncases** | **HR (95% CI)** |
| ln Cu |  | |
| Tertile 1 | 17/407 | Ref. |
| Tertile 2 | 31/396 | 1.74(0.95-3.16) |
| Tertile 3 | 47/396 | **2.34(1.33-4.13)** |
| *P*-Trend | **0.003** | |
| ln Sr |  | |
| Tertile 1 | 25/409 | Ref. |
| Tertile 2 | 29/391 | 1.40(0.81-2.41) |
| Tertile 3 | 41/399 | **1.87(1.10-3.17)** |
| *P*-Trend | **0.008** | |
| ln Pb |  | |
| Tertile 1 | 23/392 | Ref. |
| Tertile 2 | 25/403 | 1.15(0.64-2.05) |
| Tertile 3 | 47/404 | **1.84(1.11-3.09)** |
| *P*-Trend | **0.016** | |
| Bold indicates statistically significant differences (*P*<0.05).  Cox regression model was adjusted by age, sex, BMI, smoking status, diabetes, hypertension, hyperlipidemia, eGFR, peak cardiac troponin I, history of prior PCI, and stent implantation. | | |

| **Table S7. Hazard Ratios (95% CI) for All-Cause Mortality in participants with** **eGFR≥ 60 mL/min/1.73m² at baseline.** | | |
| --- | --- | --- |
| **Subgroup** | **All-Cause Mortality(n=78)** | |
|  | **Cases**  **/noncases** | **HR (95% CI)** |
| ln Cu |  | |
| Tertile 1 | 15/401 | Ref. |
| Tertile 2 | 25/395 | 1.56(0.82-3.00) |
| Tertile 3 | 38/378 | **2.40(1.31-4.41)** |
| *P*-Trend | 0.003 | |
| ln Sr |  | |
| Tertile 1 | 22/408 | Ref. |
| Tertile 2 | 27/389 | 1.40(0.79-2.50) |
| Tertile 3 | 29/377 | **1.84(1.02-3.32)** |
| *P*-Trend | 0.038 | |
| ln Pb |  | |
| Tertile 1 | 16/385 | Ref. |
| Tertile 2 | 22/390 | 1.61(0.84-3.09) |
| Tertile 3 | 40/399 | **2.19(1.21-3.97)** |
| *P*-Trend | 0.012 | |
| Bold indicates statistically significant differences (*P*<0.05).  Cox regression model was adjusted by age, sex, BMI, smoking status, diabetes, hypertension, hyperlipidemia, eGFR, peak cardiac troponin I, history of prior PCI, and stent implantation. | | |


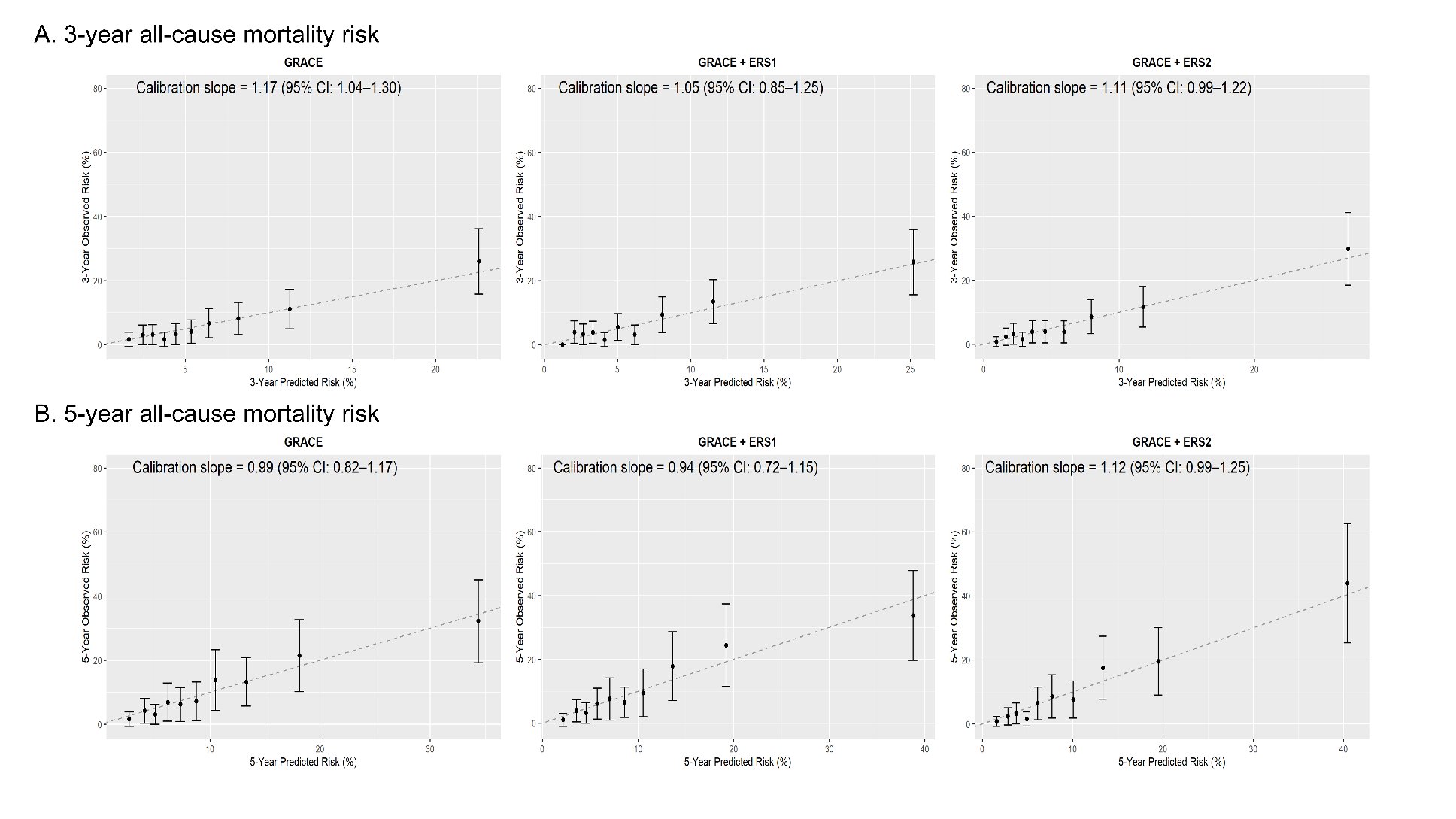


Figure S3. Observed risk is plotted against deciles of predicted risk, with 95% CIs for the observed risk and the corresponding calibration slope displayed in each panel. (A) shows 3-year and (B) shows 5-year all-cause mortality risk for GRACE, GRACE+ERS1, and GRACE+ERS2. GRACE = Global Registry of Acute Coronary Events; ERS = environmental metal risk score.


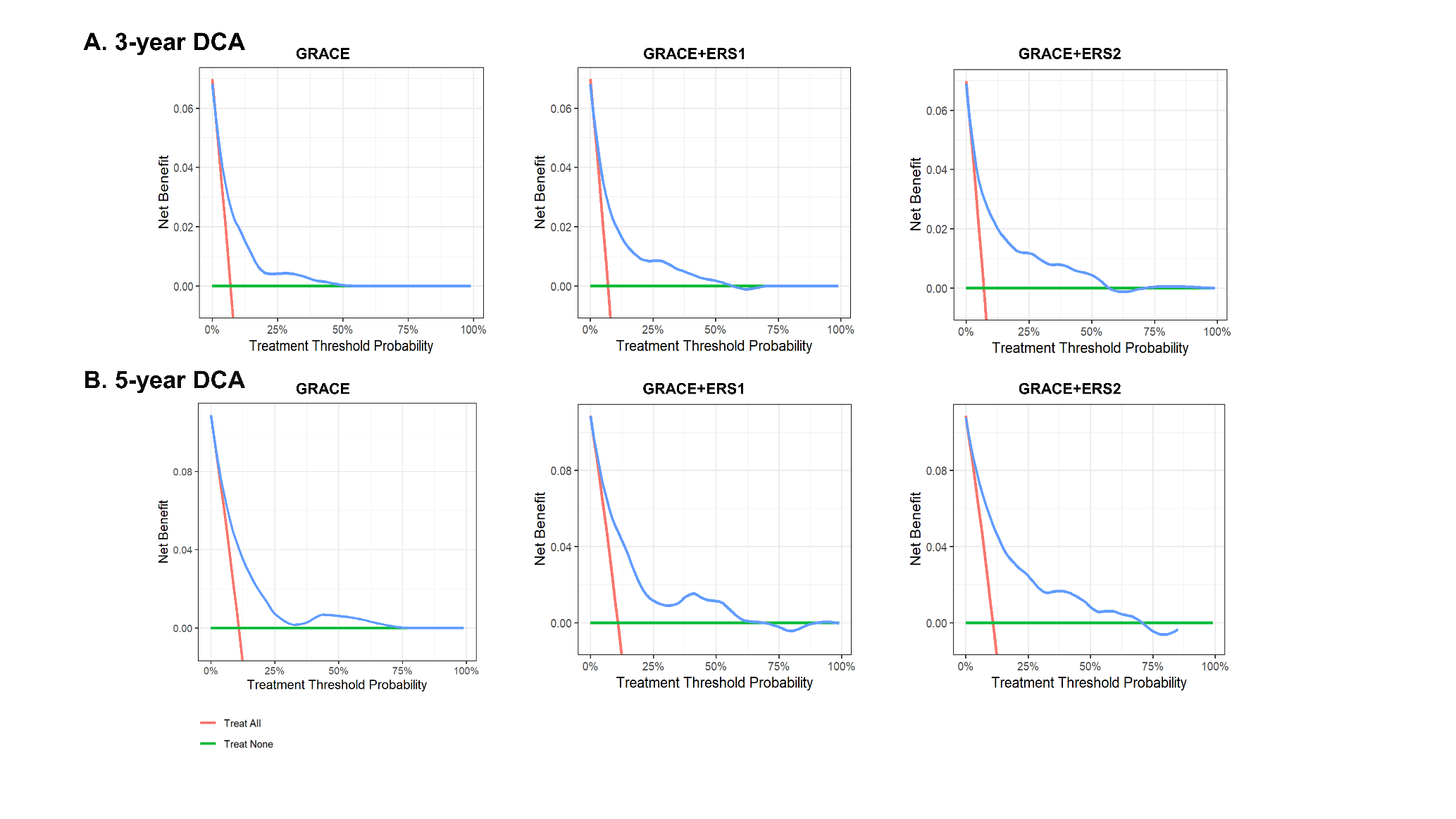


Figure S4. Decision curve analysis (DCA) for 3-year and 5-year all-cause mortality. Net benefit is plotted against the threshold probability for the GRACE, GRACE+ERS1, and GRACE+ERS2. Blue curves represent the net benefit of each prediction model, while red and green lines represent “treat all” and “treat none” strategies. GRACE = Global Registry of Acute Coronary Events; ERS = environmental metal risk score.


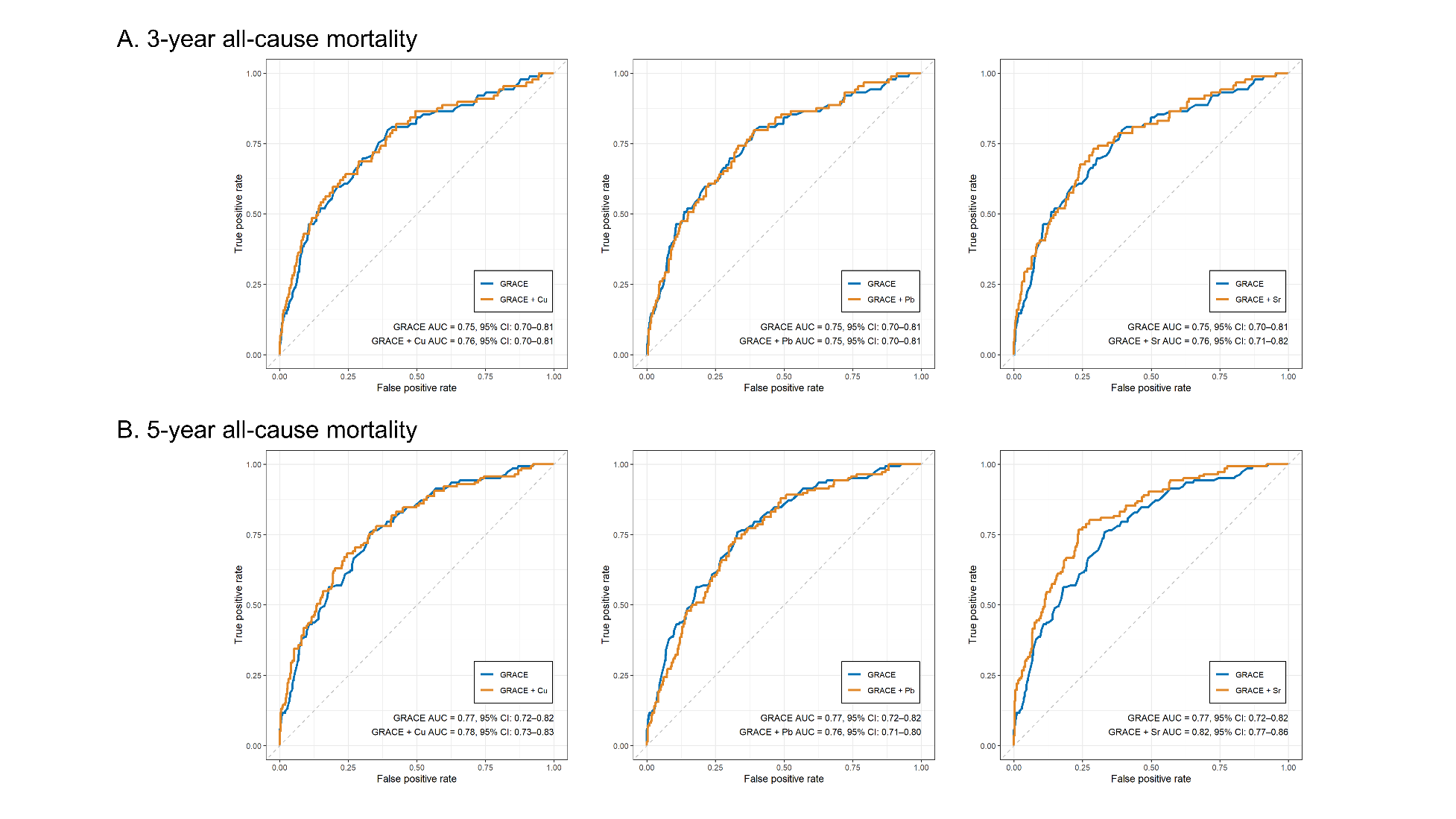


Figure S5. Time-dependent receiver operating characteristic (time-ROC) curves assessing the predictive performance of the GRACE alone and in combination with individual metals (Cu, Pb, and Sr) for 3-year and 5-year all-cause mortality. GRACE, Global Registry of Acute Coronary Events risk score; Cu, copper; Pb, lead; Sr, strontium; AUC, area under the curve.
